# Depth of anaesthesia monitoring during procedural sedation and analgesia: a systematic review protocol

**DOI:** 10.1186/s13643-015-0061-z

**Published:** 2015-05-16

**Authors:** Aaron Conway, Joanna Sutherland

**Affiliations:** Institute of Health & Biomedical Innovation, Queensland University Technology, Kelvin Grove Campus, Kelvin Grove, Queensland 4059 Australia; Cardiac Catheter Theatres, The Wesley Hospital, Auchenflower, Queensland Australia; Coffs Harbour Health Campus, Coffs Harbour, Australia; Rural Clinical School, University of New South Wales, Sydney, Australia

**Keywords:** Procedural sedation and analgesia, Conscious sedation, Deep sedation, Bispectral index, Depth of Anaesthesia monitor

## Abstract

**Background:**

Procedural sedation and analgesia (PSA) is used to attenuate the pain and distress that may otherwise be experienced during diagnostic and interventional medical or dental procedures. As the risk of adverse events increases with the depth of sedation induced, frequent monitoring of level of consciousness is recommended. Level of consciousness is usually monitored during PSA with clinical observation. Processed electroencephalogram-based depth of anaesthesia (DoA) monitoring devices provide an alternative method to monitor level of consciousness that can be used in addition to clinical observation. However, there is uncertainty as to whether their routine use in PSA would be justified. Rigorous evaluation of the clinical benefits of DoA monitors during PSA, including comprehensive syntheses of the available evidence, is therefore required. One potential clinical benefit of using DoA monitoring during PSA is that the technology could improve patient safety by reducing sedation-related adverse events, such as death or permanent neurological disability. We hypothesise that earlier identification of lapses into deeper than intended levels of sedation using DoA monitoring leads to more effective titration of sedative and analgesic medications, and results in a reduction in the risk of adverse events caused by the consequences of over-sedation, such as hypoxaemia. The primary objective of this review is to determine whether using DoA monitoring during PSA in the hospital setting improves patient safety by reducing the risk of hypoxaemia (defined as an arterial partial pressure of oxygen below 60 mmHg or percentage of haemoglobin that is saturated with oxygen [SpO^2^] less than 90 %). Other potential clinical benefits of using DoA monitoring devices during sedation will be assessed as secondary outcomes.

**Methods/design:**

Electronic databases will be systematically searched for randomized controlled trials comparing the use of depth of anaesthesia monitoring devices with clinical observation of level of consciousness during PSA. Language restrictions will not be imposed. Screening, study selection and data extraction will be performed by two independent reviewers. Disagreements will be resolved by discussion. Meta-analyses will be performed if suitable.

**Discussion:**

This review will synthesise the evidence on an important potential clinical benefit of DoA monitoring during PSA within hospital settings.

**Systematic review registration:**

PROSPERO CRD42015017251

**Electronic supplementary material:**

The online version of this article (doi:10.1186/s13643-015-0061-z) contains supplementary material, which is available to authorized users.

## Background

### Description of the condition

The aim of procedural sedation and analgesia (PSA) is to produce “a state of drug-induced tolerance of uncomfortable or painful diagnostic or interventional medical, dental or surgical procedures” (p.1) [1]. PSA is generally described as a continuum, with the risk of serious sedation-related adverse events, such as death or permanent neurological disability, increasing with the depth of sedation induced [2]. Prompt detection of lapses into deeper than intended levels of sedation is required so that corrective interventions can be implemented. For this reason, frequent monitoring of level of consciousness is recommended [1, 2]. Level of consciousness is usually monitored during PSA by clinical observation, which is performed by judging a sedated patient’s response to increasing levels of stimulation [3]. A standardised sedation assessment scale that assigns a numerical rank to observable clinical behaviours that are known to be associated with changes in the level of consciousness can be used to supplement clinical observation methods for assessing changes in level of consciousness during PSA. Processed electroencephalogram-based depth of anaesthesia (DoA) monitoring devices provide an alternative method to monitor level of consciousness that can be used in addition to clinical observation.

### Description of the intervention

The most commonly used DoA monitoring device is the bispectral index (BIS™ Covidien, Inc., Boulder, CO, USA) [4]. The device calculates a numerical derivative from brain electrical activity. It is calculated from an electroencephalogram measured at the forehead. BIS values range between 0, which represents a state of ‘no detectable brain electrical activity’, and 100, which represents the ‘awake’ state [5]. Values below 60 correspond to ‘deep’ sedation [6]. BIS monitoring during surgery with general anaesthesia results in several clinically important benefits such as reduced risk of intra-operative awareness [7], reduced anaesthetic doses and reduced recovery time [8]. Results of recent studies in patients receiving general and regional anaesthesia have suggested there may even be a survival benefit from preventing deep levels of sedation during general anaesthesia, as indicated by BIS monitoring [9, 10].

Other DoA monitors which use proprietary algorithms to process EEG information include the E-Entropy (GE Healthcare) and Narcotrend-Compact M monitors (MT Monitor Technik). The National Institute for Health and Care Excellence (NICE) has concluded that the evidence for the use of the latter two monitors is less certain than for BIS in patients receiving general anaesthesia [11]. However, the use of EEG monitors (including E-Entropy, Narcotrend-Compact M and BIS) are recommended for all patients receiving total intravenous anaesthesia [11]. Similar to the BIS, both of these monitors produce values to represent different states of the depth of anaesthesia.

### Why is it important to do this review?

The utility of DoA monitoring for sedated patients has been evaluated in a number of clinical areas within the hospital setting, such as the endoscopy suite [12] and radiology department [13]. Most of the available evidence relates to the BIS monitor. However, there remains considerable uncertainty as to whether the clinical benefits of DoA monitors justify their use [3]. Rigorous evaluation of the potential clinical benefits, including comprehensive syntheses of the available evidence, is required. To the authors’ knowledge, there are no systematic reviews on the utility of DoA monitors for procedural sedation in the published literature. Arguably, the most important potential clinical benefit of using DOA monitoring during PSA is that this technology could improve patient safety by reducing sedation-related adverse events, such as death or permanent neurological disability. We hypothesise that earlier identification of lapses into deeper than intended levels of sedation using DoA monitors leads to more effective titration of sedative and analgesic medications resulting in a reduction in the risk of sedation-related adverse events caused by over-sedation.

The frequency with which serious sedation-related adverse events occur is too low for this endpoint to be used as the primary outcome in randomized controlled trials or meta-analyses of PSA. However, it has been identified that inadequate oxygenation/ventilation is the most common reason for injury associated with sedation that is administered in the procedural setting [14]. It has been posited that surrogate outcomes must be in the causal pathway of injury with correlation alone not being sufficient [15]. A recent study reported that arterial oxygen saturation is the most important physiological parameter in assessment of risk of adverse outcomes during sedation [16]. For this reason, we determined that reduction in episodes of hypoxaemia would be the most suitable surrogate marker to investigate the potential patient safety benefits that may be associated with using DoA monitors of depth of sedation during PSA.

### Objectives

The primary objective of this review is to determine whether using DoA monitoring during PSA in the hospital setting improves patient safety by reducing the risk of hypoxaemia (defined as an arterial partial pressure of oxygen below 60 mmHg or percentage of haemoglobin that is saturated with oxygen [SpO^2^] less than 90 %). The secondary objective of this review is to synthesise the available evidence on other potential clinical benefits of DoA monitoring during sedation. Adverse effects of DoA monitoring will not be specifically examined in this review.

### Methods/design

A systematic review and meta-analyses adhering to the protocol outlined below will be conducted. Any differences between this protocol and the review will be reported.

### Criteria for considering studies for this review

#### Types of studies

Parallel and crossover randomized controlled trials comparing DoA monitoring with clinical assessment for the management of patients receiving procedural sedation and analgesia will be included. Studies that tested non-inferiority or equivalence hypotheses will be excluded because DoA monitoring is used in addition to standard monitoring not as a direct alternative. Cluster-randomised, non-randomised or quasi-randomised trials will be excluded.

#### Types of clinical settings

Studies conducted in any inpatient or outpatient setting where PSA is used in a hospital will be included. Examples of clinical settings include emergency departments, medical imaging departments, endoscopy suites and cardiac catheterisation laboratories. Dental surgeries/clinics will be excluded due to the potentially confounding influence that the different staffing profiles between these types of settings may have on clinical outcomes (i.e., the presence of a medical doctor versus a dentist).

#### Types of participants

Studies that included patients (adults or children) who received procedural sedation and analgesia (with or without local anaesthesia) will be included in the review. Studies that included patients who received general or regional anaesthesia will be excluded from the review.

#### Types of intervention

Patients whose sedation was managed by a strategy where DoA monitoring, such as bispectral index, E-Entropy and Narcotrend, was used (in addition to clinical judgement and/or a specified clinical sedation assessment tool).

#### Types of comparison

Patients whose sedation was managed without use of DoA monitoring (only clinical judgement and/or a specified clinical sedation assessment tool was used).

#### Types of outcome measures

Primary outcome:

- Hypoxaemia (arterial partial pressure of oxygen below 60 mmHg or percentage of haemoglobin that is saturated with oxygen [SpO^2^] less than 90 % for any period of time).

Secondary outcomes:

- Hypotension (systolic blood pressure less than 90 mmHg).
- Amount of sedative and analgesic medications used (a separate analysis will be undertaken for each medication).
- Duration of recovery (Defined as the time from end of procedure until Aldrete discharge recovery score more than 7. As outcomes will not form part of eligibility assessment, it will be possible to include other definitions for duration of recovery if our a-priori definition has not been reported consistently. In this circumstance, any changes between the protocol and the review will be outlined clearly in the report).
- Unable to complete procedure as it was planned due to inadequate sedation.
- Sedation-related adverse events (death, neurological deficits).

A possible mechanism of injury that could also be attenuated with improved titration of medications for PSA is organ hypoperfusion [17]. As such, we will use a standard definition for hypotension as a secondary outcome. As we hypothesise that changing clinicians’ decision-making regarding medication titration is the moderating mechanism for clinical benefits associated with DOA monitoring during PSA, a secondary outcome will be the amount of sedative and analgesic medication used. We also hypothesise that intervening at the onset of an episode of respiratory depression that would have resolved spontaneously is counterproductive and could result in inadequate sedation. For this reason, we will investigate the number of procedures needing to be abandoned due to inadequate sedation as a secondary outcome.

### Search methods for identification of studies

We will identify published, unpublished and ongoing studies by searching the following databases:

- The Cochrane Central Register of Controlled Trials (CENTRAL) (1999 (established) to present);
- MEDLINE (OvidSP) (1966 to present);
- CINAHL (EBSCOhost) (1982 to present);
- ClinicalTrials.gov; and
- World Health Organization International Clinical Trials Registry Platform.

The search strategy (Appendices 1, 2 and 3) has been designed by applying the guidance of the Cochrane Handbook for Systematic Reviews of Interventions, Chapter 6.4 [18]. Language restrictions will not be imposed for the search. If the study has been considered as potentially eligible based on the abstract, we will attempt to either have the article translated or have the required data extracted. If this cannot be done, we will include this in the report of the review.

### Data collection and analysis

#### Selection of studies

Initial screening of titles and abstracts will be performed by two independent reviewers (AC and JS). Full copies of all studies that meet the inclusion criteria will be obtained for further assessment. Second screening of full text articles will be performed by two independent reviewers (AC and JS) applying all inclusion and exclusion criteria outlined in the review protocol (Additional file 1). Disagreement on eligibility will be resolved by discussion.

#### Data extraction and management

AC and JS will independently extract data using the data extraction form (Additional file 1). Any differences of opinion will be reconciled by mutual agreement. Data will be entered into a database (Review Manager (RevMan), Version 5) for statistical analysis.

#### Assessment of risk of bias in included studies

AC and JS will undertake the risk of bias assessment of the included studies independently, as guided by the Cochrane Handbook for Systematic Reviews of Interventions [18]. The Cochrane risk of bias tool will be used to assign a judgment about the degree of risk (low risk of bias, high risk of bias and unclear risk of bias) (Additional file 1) [18].

#### Measures of treatment effect

When the measure of the outcome is sufficiently consistent across trials, we will use risk ratios for dichotomous data and mean difference or standardised mean difference for continuous data with corresponding 95 % confidence intervals (CIs).

#### Unit/scale of analysis issues

The unit of analysis is based on the individual patient (the unit that was randomised for comparison of interventions). We will use pre-crossover data for included trials that used a crossover design.

#### Dealing with missing data

It is unlikely that there will be missing data for the outcomes selected for this review, as they are measured during the procedure. A possible reason for missing data could be malfunction of monitoring equipment resulting in a loss of data about intra-procedural physiological parameters. Therefore, we will attempt to perform intention-to-treat (ITT) analyses wherever possible, and if studies do not report withdrawals, we will assume there were none.

#### Assessment of heterogeneity

To assess statistical heterogeneity we will apply the chi-squared test, as a low *p* value is evidence of the heterogeneity of intervention effects. In addition to statistical assessments, we will qualitatively review studies by examining variability in study participants, interventions and outcomes. In the absence of clinical heterogeneity, we will use the *I*^2^ statistic to describe the percentage of total variation across studies that is due to the heterogeneity rather than chance. An *I*^2^ value greater than 50 % will be considered significant heterogeneity. We will also use visual inspection of the graphical representation of study results with their 95 % CIs to assess heterogeneity.

#### Assessment of reporting biases

We will avoid publication bias by comprehensive literature searching and use of study registries [19]. We will attempt to obtain and include data from unpublished work, and no language restriction will be imposed to reduce the risk of reporting bias. We will use a graphical display (funnel plot if greater than ten studies are included) of the size of treatment effect against the precision of the trial to investigate publication bias by looking for signs of asymmetry. If there is asymmetry, we will look for reasons other than publication bias.

#### Data synthesis

First we will consider the appropriateness of undertaking meta-analysis in the presence of significant clinical or statistical heterogeneity. We will use the fixed-effect model meta-analysis except where statistical heterogeneity is identified, in which case we will use the random-effects model [18]. We will perform the meta-analyses using RevMan software (Version 5).

#### Subgroup analysis

We will consider conducting subgroup analysis if the data indicates clinical or statistical (*I*^2^ > 50 %) heterogeneity based on:

- age (adults or children);
- use of supplemental oxygen (used routinely or not);
- use of end-tidal carbon dioxide monitoring (used or not);
- the type of sedation regimen used (propofol, benzodiazepine, benzodiazepine and opioid combination, ketamine, dexmedetomidine, other)
- the type of DoA monitoring device used; and
- the type of procedure (diagnostic or interventional).

#### Summary of findings

We will present study findings in a standard ‘Summary of findings’ (SOF) table, which will include a list of the primary outcomes, a measure of the typical burden of these outcomes, the absolute and relative magnitude of effect, the numbers of participants and studies addressing each outcome and a grade for the overall quality of the body of evidence for each outcome. Space will be provided for comments. We will use the principles of the Grades of Recommendation, Assessment, Development and Evaluation (GRADE) system [20] to assess the quality of the body of evidence associated with specific outcomes (hypoxaemia, hypotension, sedation doses, duration of recovery and procedural completion) and will construct the SoF table using GRADE software. The GRADE approach appraises the quality of a body of evidence according to the extent to which one can be confident that an estimate of effect or association reflects the item being assessed. The quality of the body of evidence considers within-study risk of bias (methodological quality), directness of the evidence, heterogeneity of the data, precision of effect estimates and risk of publication bias.

## Discussion

To the best of our knowledge, this systematic review will examine, for the first time, the current state of evidence on the benefits to patient safety that may be associated with using DoA monitors instead of clinical observation to monitor level of consciousness during PSA. Reducing the risk of the most common antecedent event (hypoxia from inadequate oxygenation or ventilation) for sedation-related death and permanent neurological deficits would be a strong indicator that DoA monitors are likely to improve patient safety during PSA. For this reason, results from this systematic review will be valuable for clinical practice and research. A synthesis of the existing evidence will help clinicians who use PSA to integrate relevant findings into their daily practice. Gaps in the literature will also be identified, which could be addressed in future research.

### Additional file

Additional file 1: **Study eligibility, data extraction form and quality assessment.**

## Appendix 1: Search strategy for CENTRAL

#1 MeSH descriptor: [Electroencephalography] explode all trees

#2 (EEG or BIS or electroence*):ti,ab or (brain near monitor*) or bispectral index or e-entropy or Narcotrend or depth of anesthesia:ti,ab

#3 #1 or #2

#4 MeSH descriptor: [Conscious Sedation] explode all trees

#5 MeSH descriptor: [Deep Sedation] explode all trees

#6 #4 or #5

#7 #3 and #6

## Appendix 2: MEDLINE (EBSCOhost) search strategy

1. exp Electroencephalography/ or (EEG or BIS or electroence*).ti,ab. or (brain adj3 monitor*).mp. or “bispectral index” or e-entropy or Narcotrend or depth of anesthesis.mp.
2. Conscious Sedation/ or sedat*.ti,ab.
3. 1 and 2

## Appendix 3: CINAHL (EBSCOhost) search strategy

S1 (MH “Electroencephalography”) OR (EEG or BIS or electroence*) or (brain N3 monitor*) or “bispectral index” or e-entropy or Narcotrend or “depth of an?sthes*”)

S2 AB (sedat*) OR (MH “Conscious Sedation”)

S3 (random* or ((clinical or controlled) N3 trial*) or placebo* or prospective* or multicenter) or (blind* or mask*) N3 (single or double)

S4 S1 and S2 and S3

## Abbreviations

PSA: procedural sedation and analgesia
DoA: depth of anaesthesia
BIS: bispectral index
SOF: summary of findings
GRADE: Grades of Recommendation, Assessment, Development and Evaluation
ITT: intention-to-treat
RCT: randomized controlled trial
RevMan: Review Manager
