# Supplementary material for: Depth of anaesthesia monitoring during procedural sedation and analgesia: a systematic review protocol
Source: Syst Rev. 2015 May 16;4:70. doi: 10.1186/s13643-015-0061-z (PMC4465462; doi:10.1186/s13643-015-0061-z)
Supplement: Supplementary file 1 — Study eligibility, data extraction form and quality assessment. [file 13643_2015_61_MOESM1_ESM.docx]

| Ref. | Study | RCT  (Yes/No/Unclear) | Relevant participants  Undergoing procedure in a hospital setting with procedural sedation and analgesia  (Yes/No/Unclear) | Relevant intervention and comparison  Comparison between DoA monitor and standard monitoring without DoA monitor  (Yes/No/Unclear) | Do not proceed if any of the above answers are ‘No’. |
| --- | --- | --- | --- | --- | --- |
|  |  |  |  |  |  |

**Study Eligibility** **Data Extraction Form**

| Title of Study |  |
| --- | --- |
| Source (Journal; Year; Volume; Pages) |  |
| Authors |  |
| Not written in English |  |
| Type of Report (Full paper/abstracts/unpublished) |  |

| Characteristics of trial | |
| --- | --- |
| Country where trial was conducted |  |
| Date trial was conducted |  |
| Number of centres |  |
| Cross-over (CO), Parallel (P), Cluster (C) |  |

| Characteristics of the Participants | |
| --- | --- |
| Inclusion Criteria |  |
| Exclusion Criteria |  |
| Total number of randomised participants |  |
| total available for analysis |  |
| information on the age of the participants |  |
| information on the sex of the participants |  |
| information on the procedures |  |
| ASA status |  |
| Comorbidities (such as respiratory diseases and cardiovascular disease) |  |

| DoA monitoring group | |
| --- | --- |
| Intervention |  |
| Number of participants |  |
| additional interventions given to this group |  |

| Control Group | |
| --- | --- |
| Control |  |
| Number of participants |  |
| additional interventions given to this group |  |

| **Outcomes** | | **Reported in paper**  **(Y/N)** |
| --- | --- | --- |
| Primary | Hypoxaemia (arterial partial pressure of oxygen below 60mmHg or SpO^2^ less than 90% for any period of time) |  |
|  | *Hypoxaemia (different definition)* |  |
| Secondary | Hypotension (systolic blood pressure less than 90mmHg) |  |
|  | *Hypotension (different definition)* |  |
|  | Amount of sedative and analgesic medications used |  |
|  | Procedures not completed as planned |  |
|  | Time required to achieve recovery from sedation (defined as Aldrete recovery score >7) |  |
|  | *Recovery (different definition)* |  |
|  | Sedation-related adverse events (death, neurological deficit) |  |

| **Sugbroups** | | **Reported in paper**  **(Y/N)** |
| --- | --- | --- |
| Age | Adults |  |
|  | Children |  |
| Type of procedure | Diagnostic |  |
|  | Interventional |  |
| Supplemental oxygen | Routine |  |
|  | Not routine |  |
| End tidal carbon dioxide | Used |  |
|  | Not used |  |
| Sedation regimen | Propofol |  |
|  | Benzodiazepine |  |
|  | Benzodiazepine/opioid combination |  |
|  | Ketamine |  |
|  | Dexmedetomidine |  |
|  | Other - specify |  |
| DoA monitor | BIS |  |
|  | E-Entropy |  |
|  | Narcotrend |  |
|  | Other - specify |  |

| **For continuous data** | | | | | | | |
| --- | --- | --- | --- | --- | --- | --- | --- |
| Outcomes | | Unit of measurement | DoA group | | Control group | | Details if outcome only described in text |
|  |  |  | n | Mean (SD) | n | Mean (SD) |  |
| Secondary | Time required to achieve recovery from sedation (defined as Aldrete recovery score >7) | mins |  |  |  |  |  |
|  | *Sedation Recovery (different definition)* |  |  |  |  |  |  |
|  | Amount of sedative and analgesic medications used (add rows for each medication) |  |  |  |  |  |  |

| **For dichotomous data** | | | |
| --- | --- | --- | --- |
| Outcomes | | DoA group (n)  n = number of participants, not  number of events | Control group (n)   n = number of participants, not  number of events |
| Primary outcomes | Hypoxaemia (arterial partial pressure of oxygen below 60mmHg or SpO^2^ less than 90% for any period of time) |  |  |
|  | *Hypoxaemia (different definition)* |  |  |
| Secondary outcomes | Hypotension (systolic blood pressure less than 90mmHg) |  |  |
|  | *Hypotension (different definition)* |  |  |
|  | Proportion of procedures completed as they were proposed |  |  |
|  | Sedation-related adverse event (and add row for each specific event if possible) |  |  |

| **Other information which you feel is relevant to the results**  Indicate if: any data were obtained from the primary author; if results were estimated from graphs etc; or  calculated by you using a formula (this should be stated and the formula given). In general if results not reported in paper(s) are obtained this should be made clear here to be cited in review. |
| --- |
|  |

**Freehand space for writing actions such as contact with study authors and changes**

**Were original authors contacted? Yes/No**

**What questions were addressed?**

| Did this report include any references to published reports of potentially eligible trials not already identified for this review? | | |
| --- | --- | --- |
| First author | Journal/Conference | Year of publication |
|  |  |  |
| Did this report include any references to unpublished data from potentially eligible trials not already identified for this review? If yes, give list contact name and details | | |
|  | | |

**Quality Assessment**

| **Random sequence generation** | | | | |
| --- | --- | --- | --- | --- |
| State here method used to generate allocation and reasons for grading | | | Grade (circle) | |
| Comment on allocation by review authors or included study quote concerning allocation: | | | Low risk of bias (Random) | |
|  |  |  | High risk of bias (e.g. alternate) | |
|  |  |  | Unclear | |
| **Allocation concealment**  Process used to prevent foreknowledge of group assignment in a RCT, which should be seen as distinct from blinding | | | | |
| State here method used to conceal allocation and reasons for grading | | | | Grade (circle) |
| Comment on allocation concealment by review authors or included study quote concerning allocation: | | | | Low risk of bias |
|  |  |  |  | High risk of bias |
|  |  |  |  | Unclear |
| **Blinding** | | | | |
| Participant | High/ Low/ Unclear Risk | | | |
| Outcome assessor | High/ Low/ Unclear Risk | | | |
| Other (please specify) | High/ Low/ Unclear Risk | | | |
| Comment on blinding by review authors or included study quote concerning allocation: | | | | |
| **Intention-to-treat**  An intention-to-treat analysis is one in which all the participants in a trial are analysed according to the intervention to which they were allocated, whether they received it or not. | | | | |
| All participants entering trial |  | | | |
| Not analysed as ‘intention-to-treat’ |  | | | |
| Unclear |  | | | |

**Were withdrawals/dropouts described? Yes ?           No ?        Not clear ?**

**Number of withdrawals/dropouts**

**Reasons for withdrawals/dropouts**

**Description of withdrawals/dropouts**

**Discuss if appropriate**
